# The Association between Metabolic Syndrome and Related Factors among the Community-Dwelling Indigenous Population in Taiwan

**DOI:** 10.3390/ijerph17238958

**Published:** 2020-12-02

**Authors:** Yu-Chung Tsao, Wen-Cheng Li, Wei-Chung Yeh, Steve Wen-Neng Ueng, Sherry Yueh-Hsia Chiu, Jau-Yuan Chen

**Affiliations:** 1 Department of Occupational Medicine, Chang Gung Memorial Hospital, Linkou Branch, Taoyuan City 33305, Taiwan; eusden@gmail.com; 2 College of Medicine, Chang Gung University, Taoyuan City 33302, Taiwan; wcl20130714@gmail.com; 3 Department of Family Medicine, Chang-Gung Memorial Hospital, Linkou Branch, Taoyuan City 33305, Taiwan; sendoh777777@gmail.com; 4 Department of Health Management, Xiamen Chang-Gung Hospital, Xiamen 361028, China; 5 Department of Orthopedics, Chang Gung Memorial Hospital, Linkou Branch, Taoyuan City 33305, Taiwan; 6 Department of Health Care Management, College of Management, Chang Gung University, Taoyuan City 33302, Taiwan; sherrychiu@mail.cgu.edu.tw; 7 Department of Internal Medicine, Kaohsiung Chang Gung Memorial Hospital, Kaohsiung Branch 83301, Taiwan

**Keywords:** metabolic syndrome, substance usage indigenous people, rural health

## Abstract

The aim of this study was to conduct a community-based study with a view to construct a detailed analysis about metabolic syndrome and the related risk factors of the indigenous population. This was an observational, population-based and cross-sectional study that was conducted in remote villages of an indigenous community in northern Taiwan between 2010 and 2013. A total of 586 participants, 275 men and 311 women, were eligible for analysis. The participants underwent a questionnaire survey that included demographic and health behavior issues. An anthropometric assessment and measurements of blood pressure were carried out including serum biochemical variables. Metabolic syndrome (MetS) was defined by following the criteria provided by the modified Adult Treatment Panel III (ATP III) criteria of the National Cholesterol Education Program (NCEP). Univariate and multiple logistic regressions were used to identify the risk factors for metabolic syndrome. The standardized prevalence rates of substance use (cigarette smoking, alcohol drinking and betel nut chewing) were significantly higher than the general population regardless of whether it was northern, central or southern Taiwan and this was especially the case with betel nut chewing in women. The prevalence rate of metabolic syndrome was 42.9% in the indigenous population with 41.3% in men and 44.4% in women, which was higher than for urban Taiwanese. In the multiple logistic regression models, we found that the significant associated factors for metabolic syndrome were older age, lower education level, high levels of uric acid, alanine transaminase (ALT), gamma-glutamyl transferase (γ-GT) and creatinine. A higher prevalence rate of metabolic syndrome and substance use were observed in the indigenous population compared with urban Taiwanese, especially in women.

## 1. Introduction

Metabolic syndrome is a complex of interrelated risk factors for cardiovascular disease (CVD) and diabetes (DM). These factors include dysglycemia, raised blood pressure, elevated triglyceride levels, low high-density lipoprotein cholesterol levels and obesity (particularly central adiposity) [1,2]. Individuals with metabolic syndrome are associated with approximately five and two-fold increased risk for type 2 DM and CVD, respectively [3], as well as a risk factor for all-cause mortality [4]. Recent interest has focused on the possible involvement of insulin resistance as a linking factor although the pathogenesis remains unclear. With these risk factors, it has been demonstrated clearly that the syndrome is common and that it has a rising prevalence worldwide, which relates largely to increasing obesity and sedentary lifestyles. As a result, metabolic syndrome is now both a public health and a clinical problem. In the public health arena, more attention must be given to modification of lifestyles of the general public of all nations to reduce obesity and to increase physical activity. At a clinical level, individual patients with metabolic syndrome need to be identified so that their multiple risk factors, including lifestyle risk factors, can be reduced [1].

The prevalence of metabolic syndrome in Taiwan had been well described in recent studies [5,6]. The comparison between Taiwan and other Asian countries was also described [7]. In other countries, a higher prevalence among the indigenous population is one of the greatly emphasized public health issues [8]. However, there have been only scanty studies with small sample sizes [9,10] or a few hospital-based studies [11] that focused on the health issue of metabolic syndrome of the indigenous population in Taiwan. We aimed to conduct a community-based study with a view to constructing a detailed analysis about metabolic syndrome and related factors among an indigenous population in Taiwan. Therefore, our findings hopefully may provide valuable information for the prevention of metabolic syndrome (MetS) by further health promoting programs or intervention in this population.

## 2. Materials and Methods

### 2.1. Participants

**Figure 1:**
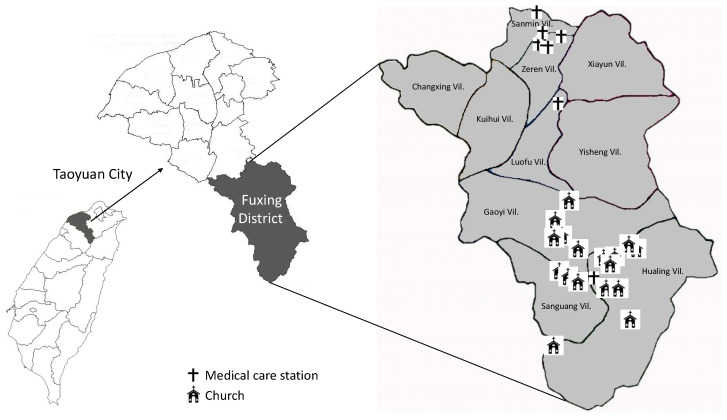
Geographical information of the remote area of the Fuxing District.

We conducted a cross-sectional study with a design based on the annual health checkup service to collect associated information between October 2010 and February 2013 from three remote villages in the Fuxing District, Taoyuan City, Taiwan (as shown in Figure 1). Participants were informed about this study and voluntarily agreed to participate. The study was approved by the Chang Gung Medical Foundation 74 Institutional Review Board (99-0231B, 101-4156A3). Participants were recruited if they were over the age of 18 and had lived in this area for more than six months. A total of 2309 residents met our inclusion criteria.

We distributed questionnaires that included basic information about personal history, occupation, substance use and exercise habits. We also arranged free health checks that included basic laboratory data. According to the data from Fuxing District Health Station, the number of actual residents who lived in the three remote villages was around 1850. Therefore, after the exclusion of potential participants with incomplete data, we had a coverage rate of 42.1% over laboratory data (*n* = 778) and 50.7% over complete questionnaires (*n* = 938). With a view to evaluating the prevalence of metabolic syndrome and related factors, we excluded those who could not be identified with the criteria of metabolic syndrome due to missing data. With these exclusion criteria, we had a coverage rate of 31.7% (*n* = 586).

Figure 1 outlines the geographic location of the mountainous area (Fuxing District) in Taoyuan city. Gaoyi, Hualing and Sanguang were the three indigenous villages where we performed the integrated delivery system. Local medical care resources and local churches were marked on the map.

### 2.2. Questionnaire

A questionnaire was used to obtain socio-demographic data (i.e., race, gender, age, education level, religion, marital status, occupation and income) as well as information on tobacco, alcohol, betel quid usage and physical activity status. Smoking, drinking and betel nut usage were categorized as either current, never or past user. Smoking and betel nut usage were defined as at least three days a week in the recent month. An alcohol drinking habit was defined as more than three times a week.

#### Definitions of Metabolic Syndrome and Measurements

Metabolic syndrome was defined by following the criteria provided by the modified Adult Treatment Panel III (ATP III) criteria of the National Cholesterol Education Program (NCEP) [3]. The presence of any three of the following five factors was required for a diagnosis of metabolic syndrome: abdominal obesity (90 cm for Asian men and 80 cm for Asian women), hypertriglyceridemia (triglycerides ≥ 150 mg/dl or current use of triglycerides-lowering drugs), low high-density lipoprotein cholesterol (HDL) (HDL cholesterol < 40 mg/dL for men and <50 mg/dL for women), elevated blood pressure (systolic blood pressure ≥ 130 mmHg and/or diastolic blood pressure ≥ 85 mmHg or current use of anti-hypertensive drugs) and impaired fasting glucose (fasting plasma glucose ≥ 100 mg/dL or current use of anti-diabetic drugs).

Blood samples were drawn from the antecubital vein following a 12-h overnight fast. After centrifugation at standard conditions, plasma samples were frozen and transported immediately to our laboratory where they were analyzed. Participants’ medical history on using anti-hypertensive and anti-diabetic medication and participants with high blood pressure or DM were recorded.

The waist circumference was measured midway between the iliac and costal margin whilst the participant was standing. The procedure used for blood pressure measurements was in agreement with the recommendations of the American Society of Hypertension, where all blood pressure measurements were conducted on the upper arm of the subject with an automatic blood pressure recorder after a 5 min resting period.

All blood analyses were performed at the clinical laboratory department of the Linkou Chang Gung Memorial Hospital and certified by the College of American Pathologists (CAP). Biochemical parameters, including high-density lipoprotein cholesterol (HDL-C), triglycerides, fasting glucose, alanine transaminase (ALT gamma-glutamyl transferase), aspartate transaminase (AST), gamma-glutamyl transferase (γ-GT), high sensitive C-reactive protein (HS-CRP), glycohemoglobulin (HbA1c), uric acid, urinary microalbumin, creatinine, low-density lipoprotein cholesterol (LDL-C) and very-low-density lipoprotein cholesterol (VLDL-C) were analyzed on a Hitachi 7600–210 autoanalyzer (Hitachi, Tokyo, Japan). Blood tests were carried out in accordance with the hospital’s laboratory standard operating procedure that was accredited by the CAP. The aforementioned biomarkers related to metabolic syndrome were selected according to previous studies [12].

### 2.3. Statistical Analysis

Descriptive statistics for demographic and biochemical variables were computed separately for male and female genders. The differences between the two groups were analyzed by t-tests or Mann–Whitney U tests of independent samples. We performed a standardized method using the WHO 2000 population [13] for prevalence comparison. The univariate and multiple logistic regressions were used for risk factor investigations. All statistical analyses were performed using SAS software version 9.4 (SAS Institute Inc., Cary, NC, USA). All tests were two tailed and a *p*-value of <0.05 was considered statistically significant.

## 3. Results

### 3.1. Metabolic Syndrome

Under the definition of the modified ATP III criteria [3], the prevalence rate of metabolic syndrome was 42.9% in the indigenous group we observed, with 41.3% in males and 44.4% in females (Table 1).

### 3.2. Substance Use

The overall crude prevalence rate of smoking was 26.4%, with 36.8% in males and 18.1% in females. The prevalence rate of alcohol drinking was 33.3%, with males 38.0% and females 29.6%. The prevalence rate of betel quid chewing was 14.1%, with males 21.3% and females 8.3%. The standardized prevalence rate of smoking, alcohol drinking and betel quid chewing were 33.3% (male 45.3%, female 25.9%), 36.7% (male 39.1%, female 35.0%) and 18.0% (male 31.9%, female 10.0%), respectively (Table 2). We chose three urban areas in Taiwan for comparison and found that the prevalence rates of substance usage were higher than in the urban area especially in alcohol drinking and betel nut chewing. The relatively higher prevalence rates of substance usage among the indigenous female population were also observed, with 25.9% smoking (9.4%, 2.5% and 2.7% in urban areas), 35.0% alcohol drinking (9.4%, 1.7% and 1.7% in urban areas) and 10.0% betel nut chewing (7.2%, 6.5% and 5.4% in urban areas) [14,15].

### 3.3. Regression Models

We built single and multiple regression models (Table 3) to discuss the related factors that are associated with metabolic syndrome. The crude odds ratio (OR) and adjusted OR are listed in Table 3. The crude ORs that were significant for metabolic syndrome were 1.03 (95% CI: 1.02, 1.04) in older age; 0.47 (95% CI: 0.31, 0.73) and 0.44 (95% CI: 0.22, 0.88) in better income; 0.48 (95% CI: 0.30, 0.76), 0.32 (95% CI: 0.19, 0.54) and 0.33 (95% CI: 0.14, 0.79) in better education; 1.93 (95% CI: 1.24, 3.02) in abnormal uric acid; 1.85 (95% CI: 1.21, 2.83) in abnormal ALT and 6.55 (95% CI: 1.86, 23.06) in abnormal creatinine. After adjusting for age and sex, the odds ratios that were significant for metabolic syndrome were 0.60 (95% CI: 0.38, 0.94) in better income, 0.46 (95% CI: 0.26, 0.83) in better education, 2.21 (95% CI: 1.22, 4.00) in betel nut chewing, 2.31 (95% CI: 1.43, 3.74) in abnormal uric acid, 1.68 (95% CI: 1.09, 2.61) in abnormal AST, 2.32 (95% CI: 1.65, 3.97) in abnormal ALT, 2.56 (95% CI: 1.65, 3.97) in abnormal γ-GT and 4.82 (95% CI: 1.33, 17.40) in abnormal creatinine. We built multiple logistic regression models and found that the models showed ORs that were significant for metabolic syndrome were 1.03 (95% CI: 1.01, 1.04) in older age, 0.44 (95% CI: 0.24, 0.83) in better education, 2.06 (95% CI: 1.22, 3.48) in abnormal uric acid, 2.02 (95% CI: 1.24, 3.29) in abnormal ALT, 1.86 (95% CI: 1.13, 3.07) in abnormal γ-GT and 5.38 (95% CI: 1.25, 23.10) in abnormal creatinine.

## 4. Discussion

### 4.1. Metabolic Syndrome

The prevalence rate of metabolic syndrome was 42.9% in the indigenous group we observed, with 41.3% in males and 44.4% in females. We performed a standardized method using the WHO 2000 population for prevalence comparison and the standardized prevalence rate of metabolic syndrome was 30.0%, with 31.9% in males and 28.3% in females. The prevalence rate was higher than that of urban Taiwanese based on recent studies with 28.6% in males and 18.8% in females [5], or similar with that of 32.4% in males and 27.8% in females [6].

A previous cross-sectional study conducted in 2008 focusing on an indigenous population (*n* = 725) in middle Taiwan showed that the prevalence rate of metabolic syndrome was 42.6%, with 41.5% in males and 43.5% in females [10]. This study showed a similar prevalence rate of metabolic syndrome with our study. Another study conducted in 2007 found that the prevalence rate of metabolic syndrome in aborigines (*n* = 90) in southern Taiwan was 83.3% (64.0% in males, 90.8% in females), which was higher than urban Taiwanese (46.6% and 40.6%, respectively) [9]. Another hospital-based study conducted in 2008 found that the prevalence rate of metabolic syndrome among indigenous people (*n* = 1226) in southeastern Taiwan was 58.7% (50.3% in males, 68.5% in females) [11].

Studies focusing on the indigenous population in other countries also showed the trend of higher prevalence rates of metabolic syndrome compared with other ethnic groups. The prevalence rate ranged from 21.4% to 37.2% in a Canadian study [8]. They suggested that the results in the indigenous community indicated that MetS was inversely associated with physical activity and fitness.

### 4.2. Substance Use

The prevalence rates for substance usage investigated by the Health Promotion Administration of Taiwan was 18.7% for smoking in 2012 and 7.8% for betel nut chewing in 2009. The prevalence rates of alcohol drinking habits among urban Taiwanese ranged from 5.6% to 8.3% [16,17,18]. The standardized prevalence rate of smoking, alcohol drinking and betel nut chewing in the indigenous group we addressed were 33.3% (male 45.3%, female 25.9%), 36.7% (male 39.1%, female 35.0%) and 18.0% (male 31.9%, female 10.0%), respectively, which were all higher than that in urban Taiwanese. Comparing the three studies from the urban cities of northern, middle and southern Taiwan, we found that the overall prevalence of substance usage was higher in this indigenous group, especially in the females.

The prevalence rate of smoking among the indigenous group has been described in previous studies with 71.1% in males and 25.2% in females [19], 58.7% in males [20] and 33.3% overall [21], which were similar results compared with our study.

The higher prevalence rate of alcohol drinking and alcohol drinking habits among indigenous groups was observed from previous studies. The results from a few studies even found that the overall prevalence rate of alcohol drinking was 85.5% in males and 58.0% in females in an indigenous group of northern Taiwan [19] and 78.6% in males in an indigenous group of middle Taiwan [20]. Another study showed that the prevalence rates of alcohol drinking among northern, southern and eastern indigenous groups in Taiwan were 22.1%, 20.3% and 25.8%, respectively [16]. These results varied due to a different definition of an alcohol drinking habit; however, a higher prevalence rate of alcohol drinking in indigenous groups was observed.

The prevalence rates of betel nut chewing were 49.7% in males and 6.3% in females in an indigenous group of northern Taiwan [19] and 48.9% in males in an indigenous group of middle Taiwan [20]. Another study showed that the prevalence rates of alcohol drinking among northern, southern and eastern indigenous groups in Taiwan were 24.1%, 33.0% and 54.2%, respectively [16]. It seems that the prevalence rate of betel nut chewing was lower than other indigenous groups and the possible reasons need further investigation.

Previous studies have proved that smoking is related to atherosclerotic CVD, neoplastic, respiratory, osteoporosis and all-cause mortality [22]. An increased risk of metabolic syndrome among current smokers, particularly those with a heavier consumption, has also been reported [23]. Alcohol drinking has also been reported as a significant contributing factor to medical conditions such as hepatitis, hypertension, tuberculosis, pneumonia, pancreatitis and cardiomyopathy [24]. Heavy drinking, in particular among liquor drinkers, is associated with an increased risk of metabolic syndrome by influencing its components [25]. A previous systemic review found that betel nut chewing was a significant risk factor for metabolic syndrome [26]. Another study found that both raw areca nuts and those with tobacco additives had a harmful relationship with metabolic syndrome [27]. Although the exact mechanism that links betel nut chewing with metabolic syndrome remains unclear, a meta-analysis proposed that the link might be related to the effect on inflammation, on adipogenesis and on appetite [28].

As the prevalence rates of metabolic syndrome and substance usage were higher than urban Taiwanese, we would like to know if substance usage was one of the confounding factors to metabolic syndrome in this indigenous group. Therefore, we built regression models to observe the trend. After adjusting for age and gender, we found that in this indigenous group, betel nut chewing was one of the confounding factors rather than smoking or alcohol drinking, which was different from previous studies. A better income and better education were indicators of a lower risk of suffering from metabolic syndrome. A cross-sectional study [10] that involved 725 indigenous residents in middle Taiwan conducted in 2008 showed similar results in that the significant confounding factors for metabolic syndrome were being aged 41–64 years old with OR = 1.81 (95% CI: 1.14, 2.88), education level at elementary school with OR = 1.61 (95% CI: 1.07, 2.42), an alcohol drinking habit with OR = 1.42 (95% CI: 0.99, 2.06) and a betel nut chewing habit with OR = 1.59 (95% CI: 1.03, 2.47).

### 4.3. Biochemical Parameters of MetS

With regard to biochemical parameters, the model was significant in the abnormal uric acid level with OR = 2.31 (95% CI: 1.43, 3.74), the elevated AST level with OR = 1.68 (95% CI: 1.09, 2.61), an abnormal ALT level with OR = 2.32 (95% CI: 1.65, 3.97), an abnormal γ-GT level with OR = 2.56 (95% CI: 1.65, 3.97) and an abnormal creatinine level with OR = 4.82 (95% CI: 1.33, 17.40).

Furthermore, the multiple logistic regression model was significant in older age with OR = 1.03 (95% CI: 1.01, 1.04), better education with OR = 0.44 (95% CI: 0.24, 0.83), abnormal uric acid levels with OR = 2.06 (95% CI: 1.22, 3.48), abnormal ALT levels with OR = 2.02 (95% CI: 1.24, 3.29), abnormal γ-GT levels with OR = 1.86 (95% CI: 1.13, 3.07) and abnormal creatinine levels with OR = 5.38 (95% CI: 1.25, 23.10). Aging has been suggested to be one of the major confounding factors for metabolic syndrome [29] and this was also proved to be one of the significant confounding factors in our study. The different prevalence rates of metabolic syndrome among men and women differs from studies [30,31,32]. Although a few studies in Taiwan have suggested a higher prevalence rate in men [33,34], we found that there was no significant difference in the indigenous population we observed. A similar finding from other indigenous populations [10] suggested that the growing prevalence among women was not only found worldwide [35] but was especially obvious in the indigenous population in Taiwan.

In this indigenous group, we observed that elevated uric acid levels, ALT levels, γ-GT levels and creatinine levels were related to metabolic syndrome. Recent studies have provided new insights into the mechanisms by which uric acid stimulates fat accumulation in the liver. Therefore, elevated serum uric acid is a strong predictor of the development of a fatty liver as well as metabolic syndrome [36]. Uric acid levels are associated with parameters of insulin resistance and indices of inflammation; however, uric acid levels do not predict the incidence of metabolic syndrome independently [37]. Another study also concluded that the elevation of ALT levels especially in obese groups reflected insulin resistance and could be a marker of metabolic syndrome [38]. A study focusing on an indigenous group also found that an elevated γ-GT level was associated with a higher waist circumference and triglyceride levels, which was also highly associated with metabolic syndrome [39]. Metabolic syndrome was suggested to be an important risk factor for chronic kidney disease [40]; however, other studies have suggested that low serum creatinine is a predictor of type 2 DM and metabolic syndrome [41].

## 5. Conclusions

Our study demonstrated that the prevalence rates of metabolic syndrome and substance use were higher in the indigenous population compared with urban Taiwanese and especially in females. Metabolic syndrome has been demonstrated to be a common precursor to the development of type 2 DM and CVD [3] as well as a risk factor for all-cause mortality [4]. We found that metabolic syndrome in this indigenous population was associated with their education level, income and biochemical parameters such as uric acid, ALT, γ-GT and creatinine levels. Therefore, we conclude that the relative lower education level, income and medical inequality could be the possible contributing factors for metabolic syndrome in this indigenous population.

This study provided us with more thorough information about the health condition and lifestyle of this indigenous group so that we can arrange further intervention programs to improve indigenous health care in clinical practice.

## Figures and Tables

**Table 1:** Background analysis of basic information, biomarkers and the prevalence of metabolic syndrome of the male and female population (*n* = 586).

Variables		Total		Male		Female		*p*-Value
		(*n* = 586)		(*n*= 275)		(*n* = 311)		(α = 0.05)
Age		49.3	±15.8	50.4	±16.9	48.4	±14.8	0.1217
Age								
	<30	65	(11.1)	33	(12.0)	32	(10.3)	0.1361
	30–39	115	(19.6)	45	(16.4)	70	(22.5)	
	40–49	118	(20.1)	53	(19.3)	65	(20.9)	
	50–59	116	(19.8)	53	(19.3)	63	(20.3)	
	60–69	104	(17.8)	50	(18.2)	54	(17.4)	
	>70	68	(11.6)	41	(14.9)	27	(8.7)	
Income (NTD, New Taiwan Dollars)								0.0028 *
	<10,000	295	(61.3)	114	(53.8)	181	(67.3)	
	10,000–29,999	140	(29.1)	69	(32.6)	71	(26.4)	
	>30,000	46	(9.6)	29	(13.7)	17	(6.3)	
	Unknown	105		63		42		
Education								0.0382 *
	≤6 grade	222	(45.9)	94	(43.9)	128	(47.4)	
	7–9 grade	135	(27.9)	51	(23.8)	84	(31.1)	
	10–12 grade	98	(20.3)	55	(25.7)	43	(15.9)	
	College or higher	29	(6.0)	14	(6.5)	15	(5.6)	
	Unknown	102		61		41		
Current cigarette smoker								<0.0001
	No	351	(73.6)	134	(63.2)	217	(81.9)	
	Yes	126	(26.4)	78	(36.8)	48	(18.1)	
	Unknown	109		63		46		
Alcohol drinking habit								0.0513
	No	320	(66.7)	132	(62.0)	188	(70.4)	
	Yes	160	(33.3)	81	(38.0)	79	(29.6)	
	Unknown	106		62		44		
Betel nut chewing habit								<0.0001
	No	410	(86.0)	166	(78.7)	244	(91.7)	
	Yes	67	(14.1)	45	(21.3)	22	(8.3)	
	Unknown	109		64		45		
DM								0.1210
	No	488	(83.3)	236	(85.8)	252	(81.0)	
	Yes (FG ≥ 126 or with history)	98	(16.7)	39	(14.2)	59	(19.0)	
Hypertension								0.9494
	No	281	(50.6)	131	(50.8)	150	(50.5)	
	Yes (SBP ≥ 140 or DBP ≥ 90 or with history)	274	(49.4)	127	(49.2)	147	(49.5)	
	Unknown	31		17		14		
Metabolic syndrome								0.4728
	No	299	(57.1)	141	(58.8)	158	(55.6)	
	Yes (ATP III Asian criteria)	225	(42.9)	99	(41.3)	126	(44.4)	
	Unknown	62		35		27		
Uric acid								<0.0001
	Normal	475	(81.1)	198	(72.0)	277	(89.1)	
	Abnormal (>8 mg/dL)	111	(18.9)	77	(28.0)	34	(10.9)	
Total cholesterol								0.0672
	Normal	326	(55.6)	142	(51.6)	184	(59.2)	
	Abnormal (≥200 mg/dL)	260	(44.4)	133	(48.4)	127	(40.8)	
High-density lipoprotein cholesterol (HDL-C)								0.0075 *
	Normal	385	(65.7)	196	(71.3)	189	(60.8)	
	Abnormal (male < 40 or female < 50 mg/dL)	201	(34.3)	79	(28.7)	122	(39.2)	
Triglyceride								0.0453 *
	Normal	345	(58.9)	150	(54.6)	195	(62.7)	
	Abnormal (≥150 mg/dL)	241	(41.1)	125	(45.5)	116	(37.3)	
Aspartate transaminase (AST)								0.2833
	Normal	463	(79.0)	212	(77.1)	251	(80.7)	
	Abnormal (>34 U/L)	123	(21.0)	63	(22.9)	60	(19.3)	
Alanine transaminase (ALT)								0.1390
	Normal	463	(79.0)	210	(76.4)	253	(81.4)	
	Abnormal (>36 U/L)	123	(21.0)	65	(23.6)	58	(18.7)	
Gamma-Glutamyl Transferase (γ-GT)								0.0019 *
	Normal	443	(75.6)	224	(81.5)	219	(70.4)	
	Abnormal (Male > 71 or female > 42 U/L)	143	(24.4)	51	(18.6)	92	(29.6)	
Creatinine								0.2333
	Normal	566	(96.6)	263	(95.6)	303	(97.4)	
	Abnormal (Male > 1.27 or female > 1.02 mg/dL)	20	(3.4)	12	(4.4)	8	(2.6)	
Hepatitis B surface antigen (HBsAg)								0.6558
	Negative	452	(82.0)	208	(81.3)	244	(82.7)	
	Positive	99	(18.0)	48	(18.8)	51	(17.3)	
	Unknown	35		19		16		
Anti-HCV antibody								0.2646
	Negative	520	(94.6)	245	(95.7)	275	(93.5)	
	Positive	30	(5.5)	11	(4.3)	19	(6.5)	
	Unknown	36		19		17		

* p-Value < 0.05.

**Table 2:** Crude and standardized prevalence of substance usage compared with other parts of Taiwan.

Substance Use	Subjects	Fuxing District		Northern Taiwan ^1^	Central Taiwan ^1^	Southern Taiwan ^1^
		Prevalence	Standardized Prevalence ^1^			
Cigarette smoking	Total	26.4	33.3	28.6	14.0	16.0
	Male	36.8	45.3	59.1	53.7	52.3
	Female	18.1	25.9	9.4	2.5	2.7
Alcohol drinking	Total	33.3	36.7	24.1	6.8	8.4
	Male	38.0	39.1	47.6	23.7	27.9
	Female	29.6	35.0	9.4	1.7	1.7
Betel nut chewing	Total	14.1	18.0	7.2	6.5	5.4
	Male	21.3	31.9	18.1	28.9	23.3
	Female	8.3	10.0	0.8	0.3	0.4

^1^ Standardized using the WHO 2000 world population, http://www.who.int/healthinfo/paper31.pdf. Total subjects: 586; male: 275, female: 311.

**Table 3:** Results of univariate and multivariate regression models on possible variants comparing those with and without metabolic syndrome.

Variables	Model 1 Univariate Regression			Model 2 Univariate Regression, Adjusted for Age and Gender			Model 3 Multivariate Regression, Adjusted for Age and Gender		
	OR	(95% CI)	*p*-Value	aOR	(95% CI)	*p*-Value	aOR (*n* = 524)	(95% CI)	*p*-Value
Gender									
Female	1.00	Ref.		1.00	Ref.		1.00	Ref.	
Male	0.88	(0.62, 1.25)	0.4728	0.81	(0.57, 1.17)	0.2587	0.76	(0.50, 1.16)	0.2029
Age	1.03	(1.02, 1.04)	<0.0001	1.03	(1.02, 1.04)	<.0001	1.03	(1.01, 1.04)	0.0006 *
Age (years old)									
<30	1.00	Ref.							
30–39	2.12	(0.96, 4.71)	0.0648						
40–49	4.99	(2.29, 10.90)	<0.0001						
50–59	5.99	(2.74, 13.11)	<0.0001						
60–69	7.47	(3.39, 16.48)	<0.0001						
≥70	4.52	(1.96, 10.45)	0.0004 *						
Income (NTD, New Taiwan Dollars)									
<10,000	1.00	Ref.		1.00	Ref.				
10,000–29,999	0.47	(0.31, 0.73)	0.0006 *	0.60	(0.38, 0.94)	0.0254 *			
>30,000	0.44	(0.22, 0.88)	0.0207 *	0.58	(0.28, 1.17)	0.1273			
Education									
≤6 years	1.00	Ref.		1.00	Ref.		1.00	Ref.	
7–9 years	0.48	(0.30, 0.76)	0.0018 *	0.68	(0.40, 1.15)	0.1538	0.59	(0.34, 1.04)	0.0676
10–12 years	0.32	(0.19, 0.54)	<0.0001	0.46	(0.26, 0.83)	0.0100 *	0.44	(0.24, 0.83)	0.0103 *
>12 years	0.33	(0.14, 0.79)	0.0126 *	0.55	(0.21, 1.41)	0.2127	0.73	(0.27, 1.97)	0.5380
Current cigarette smoker									
No	1.00	Ref.		1.00	Ref.				
Yes	1.03	(0.66, 1.61)	0.9000	1.39	(0.86, 2.23)	0.1806			
Alcohol drinking habit									
No	1.00	Ref.		1.00	Ref.				
Yes	1.30	(0.86, 1.96)	0.2081	1.51	(0.99, 2.31)	0.0558			
Betel nut chewing habit									
No	1.00	Ref.		1.00	Ref.		1.00	Ref.	
Yes	1.52	(0.87, 2.65)	0.1432	2.21	(1.22, 4.00)	0.0087 *	1.64	(0.86, 3.13)	0.1358
Uric acid									
Normal	1.00	Ref.		1.00	Ref.		1.00	Ref.	
Abnormal (>8 mg/dL)	1.93	(1.24, 3.02)	0.0039 *	2.31	(1.43, 3.74)	0.0007 *	2.06	(1.22, 3.48)	0.0071 *
Total cholesterol									
Normal	1.00	Ref.		1.00	Ref.				
Abnormal (≥200 mg/dL)	1.65	(1.16, 2.34)	0.0052 *	1.47	(1.02, 2.12)	0.0367 *			
AST									
Normal	1.00	Ref.		1.00	Ref.				
Abnormal (>34 U/L)	1.47	(0.96, 2.24)	0.0756	1.68	(1.09, 2.61)	0.0199 *			
ALT									
Normal	1.00	Ref.		1.00	Ref.		1.00	Ref.	
Abnormal (>36 U/L)	1.85	(1.21, 2.83)	0.0043 *	2.32	(1.49, 3.62)	0.0002 *	2.02	(1.24, 3.29)	0.0046 *
γ-GT									
Normal	1.00	Ref.		1.00	Ref.		1.00	Ref.	
Abnormal (Male > 71 or female > 42 U/L)	2.06	(1.36, 3.11)	0.0006 *	2.56	(1.65, 3.97)	<0.0001	1.86	(1.13, 3.07)	0.0144 *
Creatinine									
Normal	1.00	Ref.		1.00	Ref.		1.00	Ref.	
Abnormal (Male > 1.27 or female > 1.02 mg/dL)	6.55	(1.86, 23.06)	0.0035 *	4.82	(1.33, 17.40)	0.0164 *	5.38	(1.25, 23.10)	0.0237 *
Hepatitis B surface antigen (HBsAg)									
Negative	1.00	Ref.		1.00	Ref.				
Positive	0.77	(0.49, 1.23)	0.2726	0.79	(0.49, 1.26)	0.3163			
Anti-HCV antibody									
Negative	1.00	Ref.		1.00	Ref.				
Positive	1.75	(0.80, 3.81)	0.1619	1.52	(0.68, 3.37)	0.3048			

* *p*-Value < 0.05.

## Abbreviations

CVD, cardiovascular disease; DM, diabetes; ATP III, Adult Treatment Panel III; NCEP, National Cholesterol Education Program; CAP, College of American Pathologists; HDL, high-density lipoprotein cholesterol; ALT, alanine transaminase; AST, aspartate transaminase; γ-GT, Gamma-Glutamyl Transferase; HS-CRP, high sensitive C-reactive protein; HbA1c, glycohemoglobulin; LDL-C, low-density lipoprotein cholesterol; VLDL-C, very-low-density lipoprotein cholesterol.
